# Influence of the Postmortem/Storage Time of Human Corneas on the Properties of Cultured Limbal Epithelial Cells

**DOI:** 10.3390/cells11172716

**Published:** 2022-08-31

**Authors:** Gaëtan Le-Bel, Pascale Desjardins, Christelle Gross, Sergio Cortez Ghio, Camille Couture, Lucie Germain, Sylvain L. Guérin

**Affiliations:** 1 Centre de Recherche en Organogénèse Expérimentale de l’Université Laval/LOEX, and Centre de Recherche du CHU de Québec-Université Laval, Axe Médecine Régénératrice, Québec, QC G1J 1Z4, Canada; 2 Centre Universitaire d’Ophtalmologie (CUO)-Recherche, Centre de Recherche du CHU de Québec-Université Laval, Axe Médecine Régénératrice, Québec, QC G1S 4L8, Canada; 3 Département de Chirurgie, Faculté de Médecine, Université Laval, Québec, QC G1V 0A6, Canada; 4 Département d’Ophtalmologie, Faculté de Médecine, Université Laval, Québec, QC G1V 0A6, Canada

**Keywords:** cornea, tissue-engineered human cornea, microarray, postmortem/storage time, wound healing

## Abstract

Besides being a powerful model to study the mechanisms of corneal wound healing, tissue-engineered human corneas (hTECs) are sparking interest as suitable substitutes for grafting purposes. To ensure the histological and physiological integrity of hTECs, the primary cultures generated from human cornea (identified as human limbal epithelial cells (hLECs) that are used to produce them must be of the highest possible quality. The goal of the present study consisted in evaluating the impact of the postmortem/storage time (PM/ST) on their properties in culture. hLECs were isolated from the entire cornea comprising the limbus and central cornea. When grown as monolayers, short PM/ST hLECs displayed increased daily doublings and generated more colonies per seeded cells than long PM/ST hLECs. Moreover, hLECs with a short PM/ST exhibited a markedly faster wound closure kinetic both in scratch wound assays and hTECs. Collectively, these results suggest that short PM/ST hLECs have a greater number of highly proliferative stem cells, exhibit a faster and more efficient wound healing response in vitro, and produce hTECs of a higher quality, making them the best candidates to produce biomaterial substitutes for clinical studies.

## 1. Introduction

Normal homeostasis and wound healing of the corneal epithelium are ensured by corneal epithelial stem cells from the limbal area, also known as limbal stem cells (LSC). Some corneal injuries, such as chemical or thermal burns, recurrent corneal abrasion, and other inflammatory conditions, can compromise the LSC’s integrity or that of their niche [1]. In such cases, injuries may rapidly degenerate into a pathological condition named limbal stem cell deficiency (LSCD). LSCD is defined as the inability of the LSC to ensure proper renewal of the corneal epithelium due to a dysfunction or a loss of these cells, be it partial or complete [2]. Clinically, LSCD is characterized by an invasion of the cornea by the conjunctival epithelial cells, followed by neovascularization and chronic inflammation, which ultimately results in a loss of transparency and a reduction in visual acuity that may also lead to a complete loss of vision [3,4].

Unilateral LSCD may be efficiently treated with an autologous transplantation of human limbal epithelial cells (hLECs) harvested from the healthy contralateral eye and grown in vitro [5]. However, when it comes to bilateral LSCD, only an allograft of hLECs is feasible, or the use of autologous stem cells from another tissue. Allogeneic cells can be sourced from a related living donor or, more frequently, from an eye tissue bank [6,7,8]. When considering transplanting or conducting research with allogeneic cells, it is critical that hLECs with optimal proliferation, adhesion, and migration properties be used. In situ preservation parameters, i.e., tissue state and handling before cells are isolated and cultured, can obviously impact on the experimental or clinical outcomes, perhaps to a greater extent than the culture condition itself [9,10,11,12]. This is particularly true for allogeneic grafts as hLECs usually come from cadaveric corneas that may have been stored in an eye bank for a long period of time. This could also be the case for intestinal stem cells and bone marrow hematopoietic stem cells that are sometimes isolated several hours to several days after the harvest of the tissue due to delays essentially related to shipment of samples to local research facilities [13,14]. For these tissues, both postmortem time (PMT) and storage time (ST) are therefore factors of great importance. PMT corresponds to the time between the donor’s death and the tissue collection by enucleation whereas ST is the time elapsed between enucleation and the use of the corneal tissues for transplantation or cultivation. 

Three main approaches can be used for the preservation and storage of corneal tissues: cryopreservation, organ culture, and hypothermia. Although cryopreservation may be an interesting option, especially for connective tissues, corneas as well as many other epithelial tissues cannot reliably be frozen [15]. Organ culture is frequently used for corneal tissue preservation, especially in Europe. Organ culture is usually performed at 28–37 °C in DMEM supplemented with 2–8% foetal bovine serum and antibiotics, and generally allows a longer storage time of corneal tissues compared to hypothermia. Indeed, corneas can typically be stored up to 4 weeks [16], but successful transplants have also been performed with corneas conserved by organ culture up to 7 weeks [17]. Hypothermic storage of corneal explants at 2–8 °C is the most widely used approach worldwide. The principle of hypothermic storage is that the cold reduces the cell’s energy demand by decreasing its metabolism. Hypothermic maximum storage times can also be increased by using storage solutions such as Optisol-GS, which is now a widely used solution in eye banks [18]. Using this approach, storage times from 7 to up to 14 days can be achieved, with an endothelium typically better preserved than the epithelium [19].

The PMT and ST of corneal tissues can greatly vary from an eye bank to another. Some authors claim that there is no limiting factor when selecting material for corneal grafting and that there is no limit of time regarding the use of corneal tissues as long as the density of endothelial cells remains sufficient [20,21]. However, other studies have shown that a longer postmortem time also translates into a greater epithelial cell loss. According to those studies, deterioration of the corneal surface would occur as early as in the first 12 h after the donor’s death and only a ring of deep peripheral cells would remain after 5–7 days [10,22]. As such, the influence of postmortem time on clinical outcomes after a keratoplasty has not reached a consensus yet [9,23,24,25]. Moreover, no clear conclusion could be reached regarding the influence of the postmortem time on the growth rate of hLECs cultured in vitro from limbal explants as the results from these studies went both directions [26,27,28]. 

Since these results only concern explant culture, there is therefore a clear lack of literature on the subject. To our knowledge, there are no data available regarding the influence of both PMT and ST in hypothermic conditions on the proliferative potential and maintenance of hLECs in vitro following their isolation from the corneal tissue, with a majority of studies focusing only on one of these two parameters [29,30]. Yet, such data could be of great interest to ensure the validity of experimental studies and to maximize the chances of success of allogeneic cultured grafts for the treatment of patients with bilateral LSCD. In the present study, we used the term postmortem/storage time (PM/ST) to define the number of days elapsed between the death of a donor and the isolation of hLECs. Unless they are used immediately upon the death of the patient, the PM/ST therefore is the sum of both the postmortem time before harvesting the tissue, and the storage time. The aim of the present study was to investigate the impact of the PM/ST on the biological and physiological properties of hLECs. We also analyzed its influence on the ability of hLECs to regenerate a functional corneal tissue using our human-tissue-engineered cornea (hTEC) as a model.

## 2. Materials and Methods

This study was conducted in accordance with our institution’s guidelines and the Declaration of Helsinki. The protocols were approved by the CHU de Québec—Université Laval hospital and Université Laval Committees for the Protection of Human Subjects.

### 2.1. Cells Isolation and Culture

Normal human corneas unsuitable for transplantation were harvested 24 h after donors’ death (excepted for PM/ST 0 for which the cornea was obtained within 24 h following donors’ death) and obtained from our local Eye Bank (Banque d’Yeux Nationale of the Centre Universitaire d’Ophtalmologie; CHU de Québec-Université Laval Hospital, Québec, QC, Canada). Therefore, for all conditions, the postmortem time was 1 day, except for the condition PM/ST 0 where the postmortem time was 0 day. Storage time varied between 1 and 18 days, except for the condition PM/ST 0 and 1, where the storage time was 0 day. All cell populations used in this study are listed in the Supplementary Table S1. hLECs were isolated from whole human corneas, including the limbus, as previously reported [31] either directly upon reception of the corneal tissue (PM/ST-0) or at different PM/STs, ranging from 1 to 19 days. For both transportation and storage, corneas were maintained in Optisol-GS in corneal viewing chambers at 4 °C. Once isolated, the ten individual hLEC populations (PM/ST-0, -1, -2, -3, -4, -14, -15, -17, -18, and -19) were seeded in tissue culture flasks along with a lethally irradiated (6000 rad) human fibroblasts feeder layer (iHFL) and cultured in Dulbecco–Vogt modification of Eagle’s medium (Gibco, Waltham, MA, USA) with Ham’s F12 (Life Technologies, Carlsbad, CA, USA) in a 3:1 ratio (DME–HAM) supplemented with 5% Fetal Clone II serum (HyClone, Logan, UT, USA), 5 μg/mL of insulin (SAFC Bioscience, Lenexa, KS, USA), 0.4 μg/mL of hydrocortisone (Teva, Toronto, ON, Canada), 10 ng/mL of epidermal growth factor (R&D Systems, Oakville, ON, Canada), 0.212 mg/mL of isoproterenol hydrochloride (Sigma-Aldrich, Oakville, ON, Canada), 100 IU/mL of penicillin (Fresenius Kabi, Homburg, Germany), and 25 μg/mL of gentamycin (Galenova, Saint-Hyacinthe, QC, Canada) (DHc) as recently described [32,33]. Human corneal fibroblasts were isolated from the stromal portion of a cornea (from a 26-day-old donor) left after dispase digestion and the removal of both the epithelium and endothelium and cultured in Dulbecco–Vogt modification of Eagle’s medium (DME) (Gibco) supplemented with 10% foetal calf serum (HyClone, Logan, UT, USA), 100 IU/mL penicillin G, and 25 μg/mL gentamicin as previously reported [31,34]. All cells were grown under 8% CO_2_ at 37 °C and culture medium was changed every 2–3 days. 

### 2.2. Growth Rate Measurements

hLECs from each population were seeded at 5 × 10^5^ cells in 75 cm^2^ tissue culture flasks (Corning, Kennebunk, ME, USA) (T75) along with lethally irradiated iHFL (seeded at 4.5 × 10^5^ per 75 cm^2^ flask and cultured at least 7 days before the addition of hLECs). In every condition, hLECs were passaged when they reached 80–90% confluence (referred to as near-confluence). Cells were harvested at each passage (P) and counted using a Beckman Coulter counter (Z2; Beckman Coulter, Mississauga, ON, Canada) before they were seeded for the next passage. Cell counts were performed at passages P1, P2, P3, and P4 and calculated from the mean of six counts (three 75 cm^2^ flasks per condition, two counts per flask). The growth rate was determined using both the seeded and trypsinized number of cells, and the duration of culture in days according to the following formula:

$$\mathrm{Population}\;\mathrm{doublings}\;\mathrm{per}\;\mathrm{day}=\frac{\log\left(\left(\mathrm{trypsinized}\;\mathrm{number}\;\mathrm{of}\;\mathrm{cells}\right)/\left(\mathrm{seeded}\;\mathrm{number}\;\mathrm{of}\;\mathrm{cells}\right)\right)/\log\left(2\right)}{\mathrm{Duration}}$$

At each passage, cells were photographed using a Nikon Eclipse TS100 (Nikon Canada, Mississauga, ON, Canada) equipped with a numeric CCD camera (AxioCam 105 Color; Zeiss, Oberkochen, Germany), and the average cell sizes were analyzed with a Beckman Coulter sizer.

### 2.3. Colony-Forming Efficiency Assays

For the first passage (P1), hLECs from each population were thawed and seeded at 2.0 × 10^3^ cells per 25 cm^2^ tissue culture flasks (T25) containing 6.0 × 10^3^ iHFL/cm^2^. For P2 and P3, hLECs in subculture were seeded at 1.0 × 10^3^ cells in T25 containing 6.0 × 10^3^ iHFL/cm^2^. Cells were grown for 10 days under 8% CO_2_ at 37 °C and culture medium (DHc) was changed twice, after 4 and 8 days. The cultures (three T25 per condition) were fixed with a 3.7% buffered formaldehyde solution (Fisher scientific, Saint-Laurent, QC, Canada) for 30 min at room temperature and stained with 1% Rhodamine B (Sigma). Colonies, distinguished by their diameter (>4 mm (holoclones), 2–4 mm (meroclones), and <2 mm (paraclones)), were counted and the percentage of holoclones was calculated as follows:

% Holoclones = (number of colonies >4 mm/total number of colonies) × 100

### 2.4. Human Corneal Epithelial Stem Cells Count

hLECs (P1) were trypsinized (at 80–90% confluence) and fixed in suspension with PBS 1X-paraformaldehyde 3% (1.0 × 10^6^ hLECs/mL) for 10 min at room temperature. After 2 washes with PBS 1X-BSA 1%, cells were suspended at 1.0 × 10^6^ cells/mL (PBS 1X-BSA 1%) and kept at 4 °C. For cell spreads, microinserts with 4 wells (Ibidi, Gräfelfing, Germany) were deposited on charged microscopy slides (Thermo Fisher Scientific). Fixed hLECs were suspended (with PBS 1X—BSA 1%) at a final concentration of 1.0 × 10^2^ cells/µL and 10 µL of each cell suspension was added into each well (1 microinsert per hLEC population) for a total of 1.0 × 10^3^ deposited fixed cells. Charged microscopy slides with microinserts were then centrifuged 5 min at 1000× *g* and left to dry for 90 min at 37 °C. Once the wells were completely dry, the microinserts were removed from the microscopy slides and cell spreads were fixed with methanol 100% for 10 min at −20 °C, washed (PBS-IF 1X) and blocked with 10% goat serum and 0.25% triton in PBS-IF 1X for 60 min at room temperature. After washing, the samples were incubated overnight (18 h) at 4 °C with a mouse primary antibody directed against p63 (1/1, 4A4, Biocare Medical, Pacheco, CA, USA). Samples were washed with PBS-IF 1X before addition of peroxidase-conjugated AffiniPure Goat secondary antibody antimouse IgG Alexa Fluor 594 (1/200, A11005, Thermo Fisher Scientific Inc., Rockford, IL, USA) and further incubated for 60 min. All antibodies (primary and secondary) were diluted in PBS-IF 1X containing 1% goat serum. Cell nuclei were also labeled with Hoechst reagent 33258 (1/100; Sigma) following immunofluorescence staining. After washing, coverslips were mounted on glass slides with a mounting medium (glycerol, PBS, water, gelatin, and sodium azide) and kept at 4 °C before they were observed with an epifluorescence microscope (Zeiss Axio Imager.Z2 microscope; Zeiss Canada Ltd., North York, ON, Canada) and photographed with a numeric CCD camera (AxioCam MRm; Zeiss Canada Ltd.). A negligible background was observed for the controls (primary antibodies omitted). For each population, 2 cell spreads were analyzed (10 pictures per cell spread) with 3 channels: Alexa 594, Hoechst, and Brightfield. The number of cells (Hoechst), labeling intensity (Alexa 594), and cell size (Brightfield) were analyzed from pictures using CellProfiler software (Anne E. Carpenter, Thouis Jones, Lee Kamentsky, Allen Goodman, Claire McQuin, and others, Broad Institute, Cambridge, MA, USA). The percentage of human corneal epithelial stem cells was determined using both the p63 signal and cell size:

Percentage of human corneal epithelial stem cells = small cells (under 20 µm) with intense p63 nuclear staining (over 0.120)/total number of cells × 100

### 2.5. Scratch Wound Assay

hLECs (P1; 6.7 × 10^4^ cells) from each population were plated in triplicate (*n* = 3) in 9.6 cm^2^ plates with 6.0 × 10^3^ iHFL/cm^2^ in DHc. When cells reached confluence, a 1 mm × 35 mm scratch was created in the middle of the plate using a P200 pipet tip (Sarstedt, Nümbrecht, Germany). Wound closure was monitored, and photographs were captured at various time intervals (0, 5, 8, 10, and 12 h). The wound surface over time was measured using ImageJ software (Wayne Rasband, National Institute of Health, Rockville, MD, USA).

### 2.6. Production of the Human-Tissue-Engineered Corneas and Wound Healing Assays 

The two-layer hTECs were produced following the self-assembly approach as described previously [31,35,36]. Briefly, corneal fibroblasts were cultured in the presence of ascorbic acid (50 μg/mL, Sigma) for 35 days to promote the production of their own extracellular matrix. Two fibroblast sheets were then superimposed to form a reconstructed stroma, on which hLECs (P2) were seeded. Reconstructed tissues were cultured for 7 days under submerged conditions in complete DHc supplemented with ascorbate and then transferred to the air–liquid interface for 7 days in EGF-free DHc to induce epithelial differentiation. Reconstructed partial thickness corneas were then wounded using an 8 mm biopsy punch. After wounding, hTECs were placed over two additional fibroblast sheets to allow re-epithelialization over a natural extracellular matrix and culture was continued at the air–liquid interface. Wound closure was monitored macroscopically for 8 days and photographed at 24 h intervals with a Zeiss Imager Z2 microscope (Zeiss Imager.Z2 microscope (Zeiss Canada Ltd.). All experiments were conducted in quadruplicate (*n* = 4). The wound surface over time was measured using ImageJ software (Wayne Rasband).

### 2.7. Histological Analyses

Biopsy specimens from hTECs were fixed with 3.7% formaldehyde (Produits Chimiques ACP; St-Leonard, QC, Canada) and embedded in paraffin. Microtome sections (5 µm thick) were stained with Masson trichrome for histologic analysis and observed with a Zeiss Axio Imager.Z2 microscope (Zeiss Canada Ltd.). Epithelium thickness was measured by averaging the transversal length at multiple points, from pictures that covered the whole hTEC section (>15 per picture) using ImageJ software (Wayne Rasband).

### 2.8. Immunofluorescence Analyses

For immunofluorescence (IF) analyses, biopsy specimens from hTECs were embedded in optimal cutting temperature (OCT) compound (Somagen, Edmonton, AB, Canada), frozen in liquid nitrogen, and stored at −80 °C until use. Cryosections (5 µm thick) were cut using a cryostat (Leica, Concord, Canada), air-dried, fixed with acetone 100% or methanol 100% (for p63) 10 min at −20 °C, washed, and blocked with 10% goat serum or foetal calf serum (for keratin 12) and 0.25% triton (for p63) in PBS-IF 1X for 60 min at room temperature. After washing, the samples were incubated for 45 min at room temperature or overnight at 4 °C (for p63) with a mouse antibody directed against ZO-1 (1/50, 339100, Life Technologies, Grand Island, NY, USA), laminin 5 (1/200, Ab77175, Abcam, Toronto, ON, Canada), keratin (K) 15 (1/100, sc47697, Santa Cruz Biotechnology, Dallas, TX, USA), K3 (1/600, 69143, MP Biomedicals, Solon, OH, USA), Ki-67 (1/200, 556003, BD Biosciences, San Jose, CA, USA), K19 coupled Cy3 (1/200, A53-B/A2, gift from U. Karsten, Institute of Biological Sciences, University of Rostock, Germany), p63 (1/1, 4A4, Biocare Medical, Pacheco, CA, USA), a rabbit antibody directed against collagen type IV (1/200, Ab6586, Abcam, Toronto, ON, Canada), or a goat antibody directed against K12 (1/200, sc17098, Santa Cruz Biotechnology). All antibodies (primary and secondary) were diluted in PBS-IF 1X containing 1% goat serum or foetal calf serum (for keratin 12). Samples were washed with PBS-IF before addition of peroxidase-conjugated AffiniPure Goat secondary antibody antimouse IgG Alexa Fluor 488 (1/400, A11059, Thermo Fisher Scientific Inc.), antimouse IgG Alexa Fluor 594 (1/400, A11005, Thermo Fisher Scientific Inc.), antirabbit IgG Alexa Fluor 488 (1/400, A11034, Thermo Fisher Scientific Inc.), or peroxidase-conjugated AffiniPure Chicken secondary antibody antigoat IgG Alexa Fluor 594 (1/400, A21468, Thermo Fisher Scientific Inc.) and further incubated for 30 min. Cell nuclei were also labeled with Hoechst reagent 33258 (1/100; Sigma) following immunofluorescence staining. After washing, coverslips were mounted on glass slides with a mounting medium and kept at 4 °C until observation with an epifluorescence microscope (Zeiss Axio Imager.Z2 microscope; Zeiss Canada Ltd.). Tissues were photographed with a numeric CCD camera (AxioCam MRm; Zeiss Canada Ltd.). A negligible background was observed for the controls (primary antibodies omitted).

### 2.9. Western Blot

Total proteins were obtained from hLECs trypsinized (at 80–90% confluence) at P1. Each cell pellet was lysed with a TNG-T lysis buffer (15 nM NaCl, 5 mM Tris-HCl, Glycerol 1%, Triton X-100, 0.1%) supplemented with protease inhibitor cocktail (Sigma-Aldrich). Protein concentration was evaluated with the Bradford procedure. Western blots were conducted as described [37] using 5 μg (GPR75), 10 μg (PFKL), 15 μg (CEBPD), or 20 μg (ITGAM) of proteins and the following primary antibodies: mouse monoclonal antibody against CEBPD (1:100; sc365546, Santa Cruz Biotechnology), PFKL (1:100; sc393713, Santa Cruz Biotechnology), GPR75 (1:1000; sc133137, Santa Cruz Biotechnology), ITGAM (1:100; 14-0112-82, Thermo Fisher Scientific Inc.), actin (1:40,000; CLT9001, Cedarlane Laboratories Limited, Burlington, ON, Canada), and a peroxidase-conjugated AffiniPure Goat secondary antibody against mouse IgG (1:2500; 115-036-003, Jackson ImmunoResearch Laboratories, Baltimore, PA, USA). Mouse primary antibodies were incubated for 60 min at room temperature for actin, or overnight at 4 °C for CEBPD, PFKL, GPR75 and ITGAM. The secondary antibody was incubated for 90 min at room temperature. The blots were revealed using the ECL Plus Western blotting detection system (Thermo Fisher Scientific Inc.) as described [37]. The signal produced at each spot was quantified using ImageJ (Wayne Rasband). The quantification values correspond to the ratio of the signal for the protein of interest over that of actin.

### 2.10. Gene Expression Profiling

All microarray analyses were conducted by the CUO-Recherche gene profiling service (Québec, QC, Canada) as previously described [35,36]. Total RNA was obtained from the different populations of hLECs either grown as monolayers or as hTECs using the RNeasy Mini Kit (QIAGEN, Toronto, ON, Canada). All data generated from the arrays were analyzed by a robust multiarray analysis (RMA) for background correction of the raw values. They were then transformed in log2 base and quantile-normalized before a linear model was fitted to the normalized data to obtain an expression measure for each probe set on each array. Scatter plots and heat maps were generated using ArrayStar V4.1 (DNASTAR, Madison, WI, USA) software. 

### 2.11. Bioinformatics and Statistical Analyses

The ArrayStar microarray linear expression data for all hLEC populations grown as monolayers (*n* = 10) or on hTECs (*n* = 8) were uploaded into the Network Analyst, a web tool based on the R language through which they were normalized using the variance-stabilizing normalization method, and filtered to exclude low abundance (5th percentile) and low variance (15th percentile) genes. A PCA analysis was subsequently carried out with the Network Analyst to determine populations clustering. A differential gene expression analysis was then carried out between short and long PM/ST populations using the limma statistical method, which resulted in a list of statistically differentially expressed genes (adjusted *p*-value < 0.05 and logFC > 1.58). This list was then examined using the Ingenuity Pathway Analysis web-based bioinformatics application software tool (IPA; QIAGEN Inc., [38]) to compute and visualize gene interaction networks built around cellular functions of interest The maximum number of nodes from the functions of interest was set to three to limit the size of the generated interactomes, and the in silico prediction tools of IPA were used to examine how the differentially expressed genes would affect these functions. The following statistical analyses were performed using RStudio software. For holoclone analyses, scratch, and wound healing assays: the percentage of healing data was first brought to a 0 to 1 scale and then compressed. Data were then logit-transformed so that they could be fitted using linear models. Using the *nlme* package, we fitted repeated-measures linear models comprising the group (short vs. long PM/ST) and time since the beginning of the assay (or passage for the holoclone analysis), as well as an interaction term of these two variables, as fixed factors. The cell population was added as a random factor. The residual distribution and homoscedasticity were verified with Q–Q plots and residual vs. fitted values graphed, respectively. For daily doubling and cell size analyses, linear model assumptions could not be met. We thus fitted nonparametric repeated-measures linear models comprising the group (short vs. long PM/ST), passage, and an interaction term of these variables as fixed factors using the *ARTool* package. Again, the cell population was added as a random factor. For the Western blot and epithelium thickness analyses, Wilcoxon’s rank sum tests were used.

## 3. Results

### 3.1. Gene Profiling of Short and Long PM/ST hLECs Grown in Monolayer

**Figure 1:**
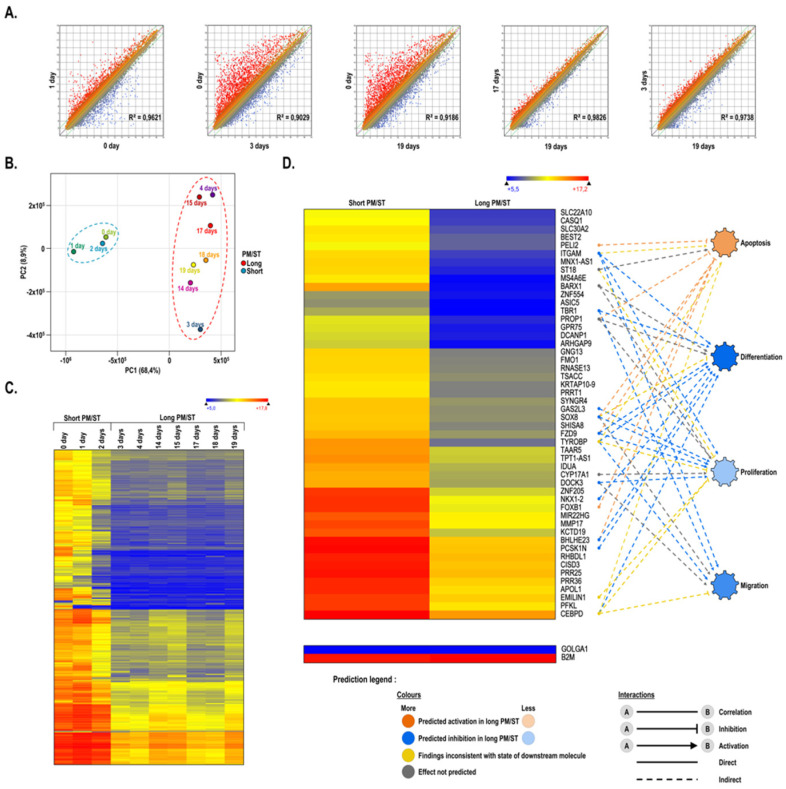
Alteration of gene expression patterns caused by the PM/ST of hLECs cultured in monolayer. (**A**) Scatter plots of log2 of signal intensity from 60,000 different targets covering the entire human transcriptome of hLECs with PM/ST 0, 3, and 19 days (*x*-axis) plotted against hLECs with PM/ST 0, 1, 3, and 17 days (*y*-axis). (**B**) Principal component analysis of gene expression patterns segregated 2 populations of hLECs: short PM/ST (_SPM/ST_hLECs) and long PM/ST (_LPM/ST_hLECs). As illustrated by the dotted ellipses, populations at 0, 1, and 2 days cluster together and differ from populations at 3, 4, 14, 15, 17, 18, and 19 days. (**C**) Heatmap representation of the 3-fold change, differentially regulated genes expressed by _LPM/ST_hLECs (long PM/ST: 3, 4, 14, 15, 17, 18, and 19 days) relative to their levels in _SPM/ST_hLECs (short PM/ST: 0, 1 and 2 days). (**D**) Heatmap representation of the 50 most differentially regulated genes expressed by _LPM/ST_hLECs relative to their levels in _SPM/ST_hLECs and their gene interaction networks based on IPA’s database and built around biological functions of interest. Data are also presented for the housekeeping genes β-2-microglobulin (*B2M*) and golgin A1 (*GOLGA1*).

We first conducted a microarray analysis to compare the gene expression profiles of hLECs with different PM/STs at passage P1. The scatter plot analyses of the 60,000 probes loaded on the array showed moderate changes in the gene expression profiles between PM/ST-0 and PM/ST-1 hLECs, and between PM/ST-17 and PM/ST-19 hLECs, as revealed by the dispersion of the normalized signals that appear as clouds of dots on Figure 1A (first and fourth panel) and the slope of the regression curve (R^2^ = 0.9621 and R^2^ = 0.9826, respectively). However, the arrays indicated that PM/ST-0 hLECs had a pattern of expressed genes distinctive from that yielded by PM/ST-3 hLECs (Figure 1A, second panel, R^2^ = 0.9029).

**Figure 2:**
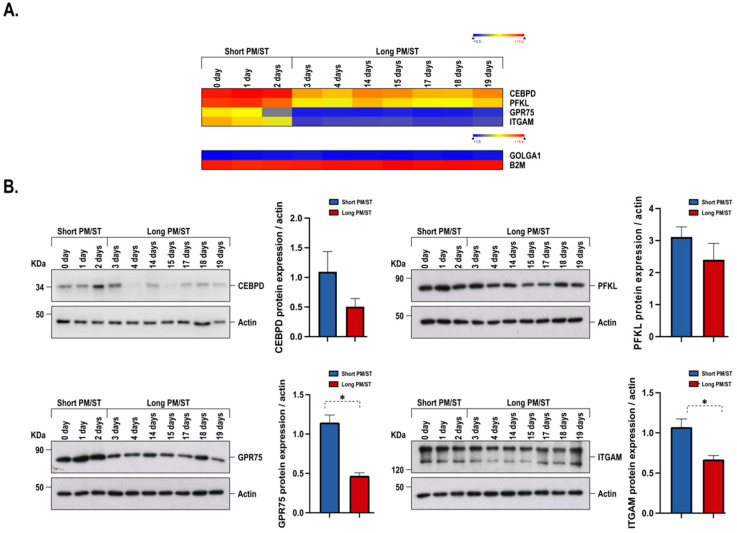
Gene expression profile and Western blot analysis of four out of the 50 most differentially regulated genes (*CEBPD*, *PFKL*, *GPR75*, and *ITGAM*) in all hLECs populations. Actin was used as the loading control. Graphs show the differences in CEBPD, PFKL, GPR75, and ITGAM expression between _SPM/ST_hLECs and _LPM/ST_hLECs. *p*-value: * < 0.08.

This expression profile was similar to the profile observed between PM/ST-0 and PM/ST-19 hLECs (Figure 1A, third panel, R^2^ = 0.9186). When both PM/ST-3 and PM/ST-19 hLECs were compared, only moderate changes were observed in the gene expression profiles (Figure 1A, last panel, R^2^ = 0.9738). These results indicate that PM/ST-3 hLECs exhibit a gene expression profile that is closer to that of PM/ST-19 hLECs rather than that of PM/ST-0 and PM/ST-1 hLECs. Considering this, we next conducted a principal component analysis (PCA; Figure 1B) in order to determine how the hLEC populations clustered. PM/ST-0, -1, and -2 hLECs clustered together and were labeled as the short PM/ST group for the remainder of the analyses. PM/STs ≥ 3 days clustered together and were labeled as the long PM/ST group. PCA analyses allowed us to separate the 10 populations of hLECs into two groups: the short PM/ST group (_SPM/ST_hLECs 0, 1, and 2) and the long PM/ST group (_LPM/ST_hLECs 3, 4, 14, 15, 17, 18, and 19). A heatmap for all the genes with at least a 3-fold gene expression variation between hLECs in the short and the long PM/ST groups was then generated (Figure 1C). Statistical analyses revealed that a total of 342 genes (*p*-values < 0.05) were significantly differentially regulated between the groups. Interestingly, Figure 1C shows that gene expression profiles were very similar within each group (short and long PM/STs) but clearly distinct between them. Figure 1D shows the 50 most differentially expressed genes (*p*-values < 0.01) between the _SPM/ST_hLECs and _LPM/ST_hLECs groups (gene expression means were calculated from all of the hLECs populations included in each group). As shown on Figure 1D, the expression of all the 50 genes identified was dramatically reduced in the _LPM/ST_hLECs group. Amongst them, several are known to encode proteins that regulate particularly important epithelial cell functions such as apoptosis (*ST18*, *CISD3*) [39,40], differentiation (*TBR1*, *BARX1*, *NKX1-2*, *FOXB1*, *APOL1*, *PROP1*, *TSACC*) [41,42,43,44,45,46,47,48,49], proliferation (*CEBPD*, *MNX1-AS1*, *GPR75*, *FZD9*, *GAS2L3*, *PFKL*, *TPT1-AS1*, *CYP17A1*, *MIR22HG*) [50,51,52,53,54,55,56,57,58], and migration (*ARHGAP9*, *ITGAM*, *EMILIN1*, *ZNF554*, *SOX8*) [59,60,61,62,63]. Moreover, the involvement of some of these genes in the cellular functions listed above was validated further by our IPA analyses. For hLECs in the long PM/ST group, we could observe an extensive decrease in the expression of genes known to regulate important epithelial processes. We next conducted Western blot analyses on four target proteins (CEBPD, PFKL, GPR75, and ITGAM) to verify whether their decreased gene expression in the microarray analysis also translated into a corresponding reduction at the protein level. As shown on Figure 2, the decreases in gene expression observed in the microarray (Figure 2A) indeed translated into a reduction in the expression of the protein encoded by these genes (Figure 2B), with a statistically significant reduction for GPR75 (*p*-value: 0.0167) and ITGAM (*p*-value: 0.0167), and a tendency observed or no statistical differences for CEBPD (*p*-value: 0.0667) and PFKL (*p*-value: 0.5167), respectively.

### 3.2. Gene Interaction Network Assisted in Silico Predictions of Biological Functions Regulation in Short and Long PM/ST hLECs Cultured in Monolayers

The data from the 274 statistically differentially expressed genes between both the short and long PM/ST groups (adjusted *p*-value < 0.01 and logFC > 1.58) were next uploaded into Ingenuity Pathway Analysis (IPA) software to be further analyzed. IPA’s statistical algorithms and curated knowledge database can be used to predict how and which biological functions are likely to be influenced when provided with data from a differential expression analysis. We thus selected several biological functions of interest (apoptosis, proliferation and differentiation of epithelial cells, colony formation and wound healing, and proliferation of stem cells) to which we connected all the genes that were linked to these functions according to the database, but that were also significantly differentially expressed in our dataset (Supplementary Figure S1). The maximum number of nodes from the functions of interest was set to three. We then examined how these genes interacted and computationally predicted how the resulting networks affected the biological functions of interest. Given our microarray data analysis, IPA predicted that _SPM/ST_hLECs would proliferate more than _LPM/ST_hLECs but differentiate less. An increase was also predicted for colony formation, wound healing, and stem cell proliferation in the _SPM/ST_hLECs, contrary to apoptosis, which was increased in the _LPM/ST_hLECs. Some of the 50 most differentially expressed genes were also recognized by IPA to impact on the different cellular processes analyzed. Indeed, CEBPD and ITGAM are known to be involved in apoptosis, proliferation, and the differentiation of epithelial cells, as well as wound healing and the proliferation of stem cells. As for PFKL and GPR75, they are involved in colony formation and stem cell proliferation.

### 3.3. Growth Rate, Colony-Forming Efficiency, Cell Size, Morphology, and Stem Cell Analysis of Short and Long PM/ST hLECs Grown in Monolayer

**Figure 3:**
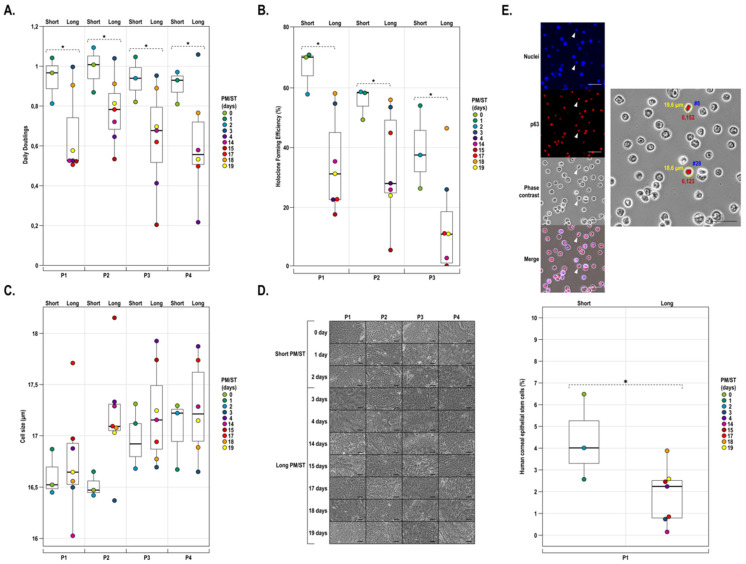
Proliferative properties, holoclone-forming capacity, cell size, morphology, and stem cell analysis of hLECs with short or long PM/ST. (**A**) hLECs isolated from donor eyes with different PM/STs (short PM/ST: 0, 1, and 2 days; long PM/ST: 3, 4, 14, 15, 17, 18, and 19 days) were cultured until P4. Graph representation of the average growth rates calculated as the number of population doublings per day. (**B**) Graph representation of the holoclone-forming percentage calculated from the colony-forming cells present in the different hLECs populations cultured at P1, P2, and P3. The number of holoclones can be used as a surrogate for the evaluation of the number of stem cells contained in the culture. (**C**) Average cell size in μm. (**D**) Morphology by phase contrast microscopy of hLECs cultured at passage P1 to P4. Scale bars: 200 µm. (**E**) Top panel: One microscopic field showing the detection of stem cells on spread cells. Left pictures are nuclei stained with Hoechst, p63, phase contrast, and merging of the last two (stem cells are identified by arrowheads). On the right, detection of two corneal epithelial stem cells (shown in red) and their respective size (indicated in yellow) by picture analysis of fluorescence intensity of p63 labeling with CellProfiler software (staining > 0.120, size < 20 µm). Bottom panel: graph representation of the percentage of corneal epithelial stem cells in different hLECs populations at P1. Scale bars: 50 µm. *p*-value: * < 0.08.

HLECs were cocultured in a monolayer with iHFL in DHc in order to evaluate the PM/ST impact on the growth rate, colony-forming efficiency, size, and morphology of these cells. As hLECs are usually cultured for up to two or three passages when used for clinical purposes, we subcultured them until they reached P4. As shown on Figure 3A, the growth rate was affected by the PM/ST at all passages. Indeed, _SPM/ST_hLECs displayed increased daily doublings when compared with _LPM/ST_hLECs (Figure 3A). A strong tendency could be detected (*p*-value: 0.0523) and those differences were maintained over passages in culture (P1 to P4).

Colony-forming efficiency (CFE) was also evaluated at passages P1, P2, and P3. _SPM/ST_hLECs generated more colonies per seeded cell than _LPM/ST_hLECs (Figure 3B). In addition, the holoclone percentages for _SPM/ST_hLECs relative to _LPM/ST_hLECs were higher at all passages_._ A tendency could be detected (*p*-value: 0.0742) and differences were maintained over passages in culture (P1 to P3). Cell size and morphology can be important biomarkers of the differentiation state and proliferative potential. Indeed, less differentiated hLECs are characterized by a smaller size and a high nucleus-to-cytoplasm ratio while differentiated hLECs have a smaller nucleus-to-cytoplasm ratio. They were present in the cultures together with colonies comprising more differentiated cells. Given that the epithelial cell size increases upon terminal differentiation [64], the average hLECs size was analyzed for the 10 populations at each passage. Figure 3C shows that the average cell size suddenly increased at P3 for the _SPM/ST_hLECs and P2 for the _LPM/ST_hLECs. With the only exception of cells at P2, no statistically significant differences were detected in the cell size between the _SPM/ST_hLECs and _LPM/ST_hLECs groups at all passages (*p*-value > 0.1). No obvious differences were observed in the cell morphology between hLECs with different PM/STs over cell passages (P1 to P4; Figure 3D and Supplementary Figure S2), except for _LPM/ST_hLEC-4 and _LPM/ST_hLEC-15 hLECs, which displayed a more elongated shape characteristic of a fibroblastic morphology. More importantly, there was no obvious morphological differences between _SPM/ST_hLECs and _LPM/ST_hLECs. We next evaluated the percentage of corneal epithelial stem cells in each of the ten hLEC populations. Two main criteria were used for the identification of stem cells in hLEC spreads using CellProfiler software, the cell size and the intensity of the p63 immunostaining. As shown on Figure 3E, _SPM/ST_hLECs yielded a statistically significant higher percentage of stem cells (4.35%) compared to that obtained for _LPM/ST_hLECs (1.84%) (*p*-value: 0.025).

### 3.4. Wound Closure of Short and Long PM/ST hLECs Grown in Monolayer 

**Figure 4:**
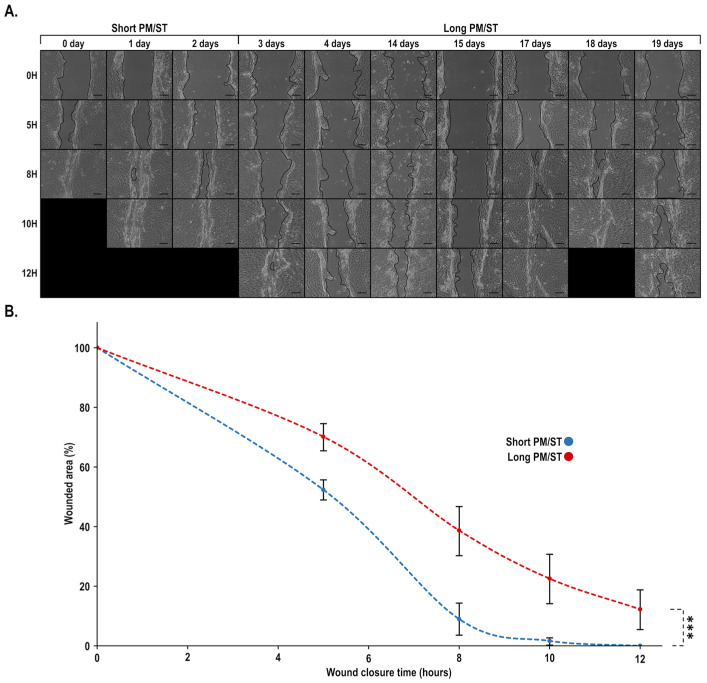
Impact of PM/ST on wound closure of hLECs grown in monolayer. (**A**) hLECs with different PM/STs grown as a monolayer were wounded and allowed to migrate until 12 h postwounding. Scratches (four per condition) were photographed at various time intervals (0 to 12 h) to monitor wound closure. (**B**) Wound surfaces remaining for each condition were determined at various time intervals (0, 5, 8, 10, 12 h) and plotted on a graph. Scale bar: 200 μm. *p*-value: *** < 0.001.

As no study ever examined the impact of the PM/ST on the dynamic of corneal wound healing, hLECs were grown as monolayers and scratch-wounded. Figure 4A shows that several wounded monolayers of cells from the _LPM/ST_hLECs group (_LPM/ST_hLEC-4, -14, -15, and -19 days) suffered from a severely delayed wound closure as most damages had not completely closed at 12 h postinjury. Statistically significant differences in the wound closure kinetic were observed between both conditions (*p*-value: 0.0005) and were maintained over time (for 5, 8, 10, and 12 h; Figure 4B). This can be explained by the fact that all _SPM/ST_hLECs populations (_SPM/ST_hLEC-0, -1, and -2 days) were completely healed between 8 and 10 h, in comparison with _LPM/ST_hLECs, which, for most of them, needed more than 12 h for a complete wound closure to occur.

### 3.5. Morphology and Thickness of the Stratified Corneal Epithelium When Short and Long PM/ST hLECs Are Used for the Production of Human-Tissue-Engineered Corneas (hTECs)

**Figure 5:**
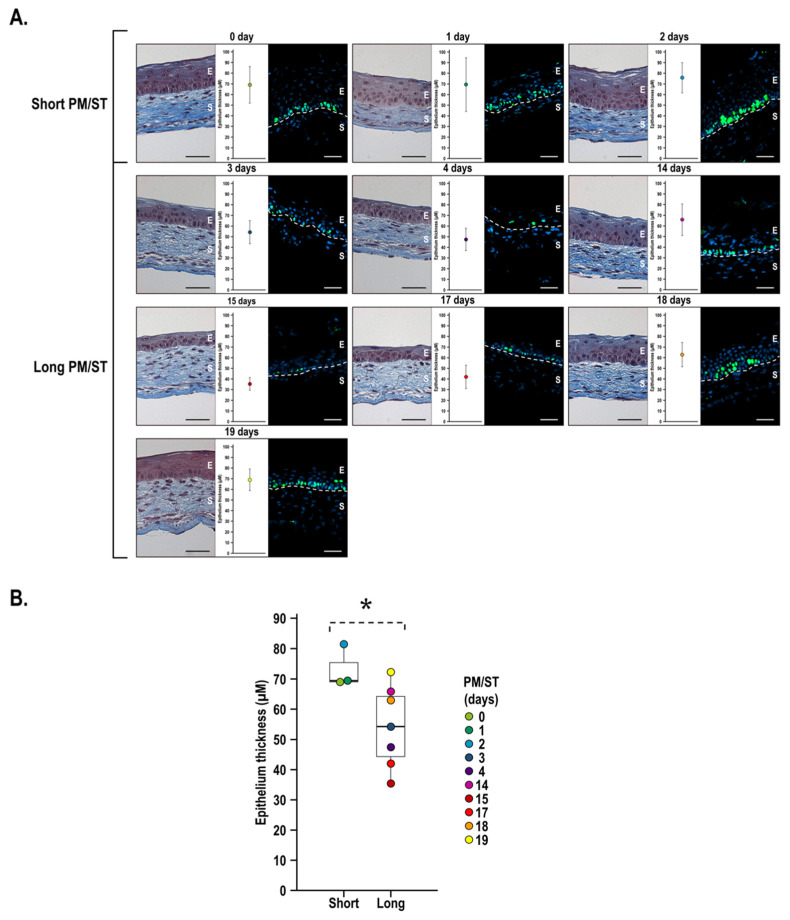
Analysis of morphology and thickness of the stratified corneal epithelium when short and long PM/ST hLECs are used to produce human-tissue-engineered corneas (hTECs). Human-tissue-engineered corneas (hTECs) were reconstructed by seeding hLECs with various PM/STs over reconstructed stroma to produce a 3D construct. (**A**) Histology (Masson’s trichrome staining), epithelium thickness (µm), and Ki-67 (immunofluorescence) analyses of _SPM/ST_hTECs (short PM/ST: 0, 1, and 2 days) and _LPM/ST_hTECs (long PM/ST: 3, 4, 14, 15, 17, 18, and 19 days). (**B**) Graph of mean epithelium thickness of _SPM/ST_hTECs (short) and _LPM/ST_hTECs (long). E: epithelium; S: stroma. Scale bars: 50 µm. *p*-value: * < 0.08.

In order to determine whether _LPM/ST_hLECs were as effective as _SPM/ST_hLECs to generate a stratified corneal epithelium in 3D human-tissue-engineered corneas (hTECs), we cultured all populations of hLECs and then used these epithelial cells to produce hTECs (_SPM/ST_hTECs and _LPM/ST_hTECs) by the self-assembly approach. After maturation at the air–liquid interface for 7 days (to allow the stratification of the epithelium), the corneal epithelia were composed of four to seven cell layers (Figure 5A). Representative histological cross sections showed that well-stratified corneal epithelia possessed cuboidal basal cells, which flattened as they differentiated into superficial cells for all populations of hLECs. Epithelia from _SPM/ST_hTECs had more cell layers than some of the _LPM/ST_hTECs (Figure 5A). The expression of Ki-67 was also assessed as a proliferation marker of hLECs. Unsurprisingly, Ki-67 labeled a greater number of basal cells in hTECs with the thickest epithelia (_SPM/ST_hLEC-0, -1, -2 and _LPM/ST_hLEC-14, -18, and -19 days). Thickness measurements of the hTECs ranged from 35 to 72 µm depending on the PM/ST (Figure 5B). One general observation was that some of the hTECs in the _LPM/ST_hTECs group showed a thinner epithelium than those in the _SPM/ST_hTECs group, which also appeared to be associated with a decreased expression of Ki-67. However, no statistically significant difference but a tendency could be observed between _SPM/ST_hTECs and _LPM/ST_hTECs (*p*-value: 0.0667; Figure 5B).

### 3.6. Integrity of the hTECs Produced Using Short and Long PM/ST hLECs

**Figure 6:**
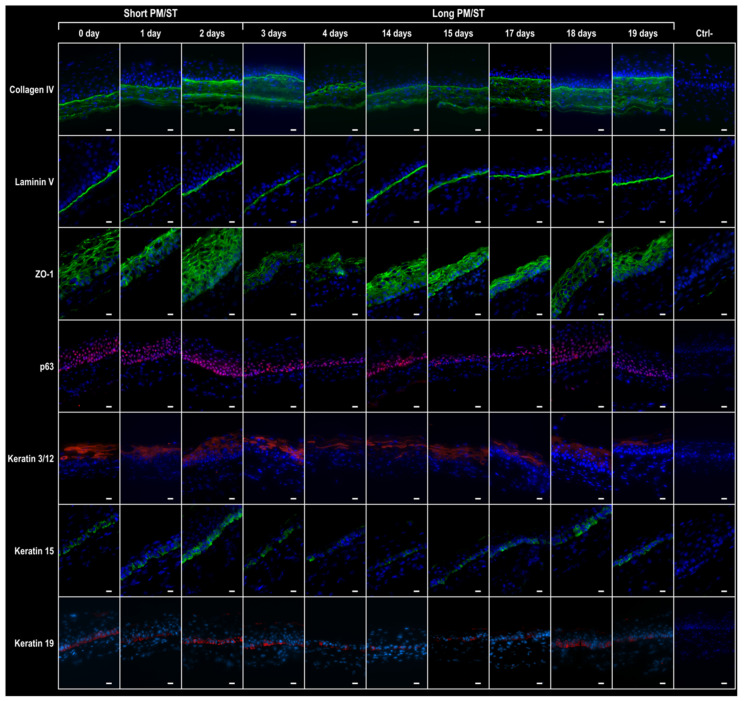
Immunofluorescence analysis of human-tissue-engineered corneas (hTECs) produced using hLECs with short and long PM/ST. Human-tissue-engineered corneas (hTECs) were reconstructed by seeding hLECs with various PM/STs over reconstructed stroma to produce a 3D construct. Immunofluorescence staining of epithelial basement membrane components (collagen IV and laminin 5), corneal epithelial cell junctions (ZO-1) epithelial tissue development regulator (p63), differentiation marker for corneal epithelial cells (K3 and K12), and poorly differentiated corneal epithelial cells (K15 and K19) in both _SPM/ST_hTECs (short PM/ST: 0, 1, and 2 days) and _LPM/ST_hTECs (long PM/ST: 3, 4, 14, 15, 17, 18, and 19 days). Ctrl-: immunofluorescence without primary antibody. Scale bar: 20 µm.

After maturation at the air–liquid interface for seven days, several indirect immunofluorescence assays were conducted to evaluate the integrity of the epithelial compartments of _SPM/ST_hTECs and _LPM/ST_hTECs (Figure 6). The secretion and localization of laminin 5 and collagen type IV, two major components from the corneal basement membrane, was evaluated. Both basement membrane components were present as a continuous line along the epithelium–stromal junction for all populations. The expression of ZO-1, a typical cellular junction protein, was also detected in all the reconstructed epithelia irrespective of the hLEC population used in their production. Unlike ZO-1, whose expression was similar between all conditions, the expression of p63, an important factor regulating epithelial tissue development, was decreased in _LPM/ST_hLEC-4, -15, and -17 hTECs. The K3 and K12 keratin pair, a well-known marker of differentiated corneal epithelial cells, was detected in all hTECs but to a lesser extent in _LPM/ST_hLEC-4, -17, -18, and -19 hTECs. Staining for K15 and K19, both known as markers of poorly differentiated corneal epithelial cells, was detected in all hTECs but a lower expression was observed in _LPM/ST_hLEC-4, -14, -15, -17, and -19 hTECs. We therefore conclude that certain tissues in the _LPM/ST_hTECs group also have a decreased expression of different important factors for the implementation of a stratified corneal epithelium.

### 3.7. Wound Closure Dynamic of hTECs Produced Using Short and Long PM/ST hLECs

**Figure 7:**
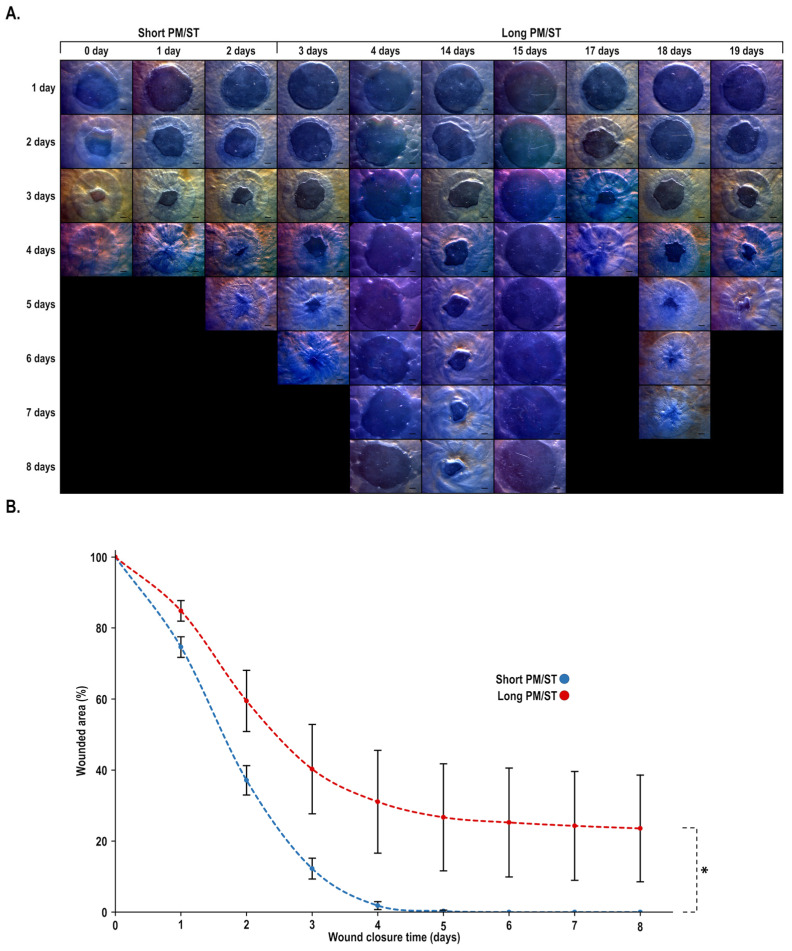
Impact of hLECs PM/ST on human-tissue-engineered corneas (hTECs) wound closure. Human-tissue-engineered corneas (hTECs) were reconstructed by seeding hLECs with various PM/STs over reconstructed stroma to produce a 3D construct. (**A**) Both _SPM/ST_hTECs (short PM/ST: 0, 1, and 2 days) and _LPM/ST_hTECs (long PM/ST: 3, 4, 14, 15, 17, 18, and 19 days) were wounded using an 8 mm biopsy punch and allowed to recover in culture at the air/liquid interface. Corneas were photographed each day (days 0 to 8) to monitor wound closure. (**B**) Graph of the percentage of wound surface area remaining as a function of time for each condition (*n* = 4). Scale bar: 1 mm. *p*-value: * < 0.05.

We next produced 8 mm biopsy-punched wounds on both SPM/SThTECs and LPM/SThTECs in order to evaluate the impact of the PM/ST on the dynamic of wound closure. Wounds were monitored macroscopically until complete closure. As shown on Figure 7A, complete wound closure was reached in 4–5 days in the SPM/SThTECs, whereas the LPM/SThTECs required up to 8 days to heal completely or partially for some of them. Wound closure kinetics (Figure 7B) showed a statistically significant difference between both conditions (*p*-value: 0.0484) and that difference was maintained over time (1, 2, 3, 4, and 5 days).

### 3.8. Gene Profiling on Microarrays of hTECs Produced Using Short and Long PM/ST hLECs

**Figure 8:**
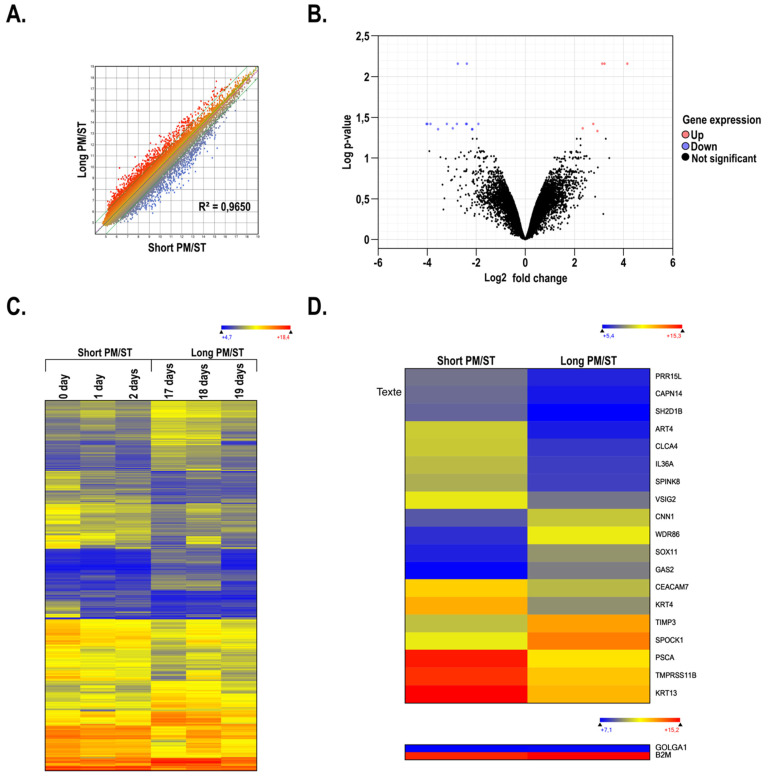
Gene expression analysis of human-tissue-engineered corneas (hTECs) produced using hLECs with short and long PM/STs. (**A**) Scatter plots of log2 of signal intensity from 60,000 different targets covering the entire human transcriptome of _SPM/ST_hTECs (short PM/ST: 0, 1, and 2 days; *x*-axis) plotted against _LPM/ST_hTECs (long PM/ST: 3, 4, 14, 15, 17, 18, and 19 days; *y*-axis). (**B**) Volcano plot of gene expression differences between _SPM/ST_hTECs and _LPM/ST_hTECs. Black dots represent genes that are not significantly differentially expressed between the two conditions. Red and blue dots indicate genes that are up- or downregulated, respectively, in _SPM/ST_hTECs when compared to _LPM/ST_hTECs. (**C**) Heatmap representation of the 3-fold change differentially regulated genes expressed by _LPM/ST_hTECs (long PM/ST: 17, 18, and 19 days) relative to their levels in _SPM/ST_hTECs (short PM/ST: 0, 1, and 2 days). (**D**) Heatmap representation of the 19 most differentially regulated genes expressed by _LPM/ST_hTECs (data shown are the mean of long PM/ST: 17, 18, and 19 days) relative to their levels in _SPM/ST_hTECs (data shown are the mean of short PM/ST: 0, 1, and 2 days). Data are also presented for the housekeeping genes β-2-microglobulin (B2M) and golgin A1 (GOLGA1).

We next conducted gene expression profiling on microarrays using total RNA prepared from hTECs constructed using _SPM/ST_hLEC-0, -1, and -2 and _LPM/ST_hLEC-17, -18, and -19 hLECs and compared the pattern of expressed genes between those assembled using hLECs with either short (_SPM/ST_hTECs) or long (_LPM/ST_hTECs) PM/STs. Scatterplot analyses revealed restricted changes in the pattern of genes expressed by _LPM/ST_hTECs relative to _SPM/ST_hTECs as indicated by the minor variations noted in the slope of the regression curve (Figure 8A; R^2^ = 0.9650). A volcano plot showing a threefold (Log FC 1.58) or more expression variation between _SPM/ST_hTECs and _LPM/ST_hTECs was then generated (Figure 8B). Consistent with the data from the scatter plot analysis, only 19 genes, with *p*-values < 0.05, fitted into that category of differentially regulated genes between both types of hTECs. A heatmap for all the genes showing a threefold or more gene expression variation between _SPM/ST_hTECs and _LPM/ST_hTECs was then generated (Figure 8C). Unlike the data yielded when hLECs were grown in monolayers (Figure 1C), the pattern of genes differentially regulated by more than threefold was clearly more uniform between _SPM/ST_hTECs and _LPM/ST_hTECs. We then examined the data files from the microarrays to sort out the most differentially regulated genes (*p*-values < 0.05) between _SPM/ST_hTECs and _LPM/ST_hTECs (Figure 8D). Of the 19 genes identified as differentially expressed between the two groups, several are known to encode products that influence hLECs migration, proliferation, and extracellular matrix remodelling (*SPOCK1*, *TMPRSS11B*, *CLCA4*, *SPINK8*, *CAPN1*, *IL36A*, and *PSCA*) [65,66,67,68,69,70,71]. In addition, 2 of these 19 genes encode keratins (*KRT4*, *KRT13*) whose respective expression was considerably higher in hTEC_sPM/ST_ than in hTEC_lPM/ST_.

## 4. Discussion

The unilateral LSCD syndrome can be efficiently treated with an autologous cultured hLECs transplant [5,72]. However, the transplantation of allogeneic cells remains one of the few options to treat patients with bilateral LSCD. Allogeneic cells come from cadaveric corneal tissue preserved in an ocular tissue bank, especially when there is no related and compatible donor available. Under these conditions, it is possible that the PM/ST of hLECs, defined as the time elapsed from donor death to hLECs isolation and culture, could influence their growth. It is well known that allogeneic procedures have a higher failure rate than autologous transplantations [73]. It is therefore important to determine whether the PM/ST can impact the ability of hLECs to proliferate and influence the success or failure of transplantation. In this study, we demonstrated that the PM/ST could influence the proliferation of hLECs in culture. An increase in the PM/ST was associated with a decrease in the proliferation, clonogenicity, and stem cell percentage of hLECs grown in a monolayer. An increase in the PM/ST was also associated with a reduction of both the epithelium thickness and the wound closure dynamic in our hTEC model. 

Most clonogenic hLECs act as transient progenitors, therefore suggesting that the growth rate and CFE percentage is insufficient to evaluate the efficiency of our hLECs culture [74]. Therefore, a rapid evaluation of the stem cell subpopulation within hLECs cultures is an interesting avenue. Pellegrini’s team developed a method, based on the detection of the p63 transcription factor by immunostaining, to quantify corneal epithelial stem cells so that the clinical success of the patient graft can be predicted [5,75]. Indeed, cultures containing more than 3% of stem cells had a patient transplant success rate of at least 80%, whereas those with less than 3% stem cells had a success rate of only 11% [5]. For the purposes of our study, we adapted this procedure to better characterize our hLECs cultures. Based on our p63 immunofluorescence analyses, we determined that the _SPM/ST_hLECs contained 4.35% of stem cells compared to 1.84% for _LPM/ST_hLECs. These results could explain why the _SPM/ST_hLECs also exhibited a faster healing in our scratch assay (monolayer model) and wound healing experiments (hTEC 3D model). The higher proportion of holoclones indicated a higher percentage of stem cells in the _SPM/ST_hLECs that could also explain their increased growth rate, as well as the fact that hLECs from this group could generate a thicker epithelium when seeded on the reconstructed stroma. 

Previous studies suggested that structural changes in the epithelium were observed with an increasing PMT [22]. Indeed, a decrease in the in situ number of suprabasal cells of the limbal epithelium was observed 5 to 7 days after a donor’s death, leaving only the basal cell layer. These observations are in agreements with the fact that 24 to 48 h after death, the deterioration of the corneal structure is accompanied by the disappearance of almost all of the corneal epithelium in 70% of cases [10]. This explains in particular the fact that a longer PMT is associated with the presence of corneal epithelial defects in the early postoperative period after the transplant [9,12,23]. These results can be explained by the fact that in the absence of suprabasal cells, the cells from the basal layer of the limbal epithelium where the LSC niche resides [76] are further exposed, which makes them more sensitive to biopsy manipulations, environmental changes imposed by extraction, or storage conditions. LSCs, like a large number of stem cells, are very dependent on the integrity of their niche to function properly [77]. Moreover, since vascular perfusion of the niche ceases upon the death of the donor, the time between death and transfer of the corneal tissue in the nutrient environment of the storage solution may affect the survival of LSCs. All of this may explain that corneal tissues with a very short PMT were significantly more likely to lead to a viable culture of hLECs in vitro [26,27]. These data agree with our own observations, which showed that the smallest PMT 0-day population presented the highest percentage of stem cells with one of the best growth rates, holoclone percentages, and wound closing rates. However, viable hLECs have been shown to still reside in the epithelium of donor corneas up to 7 days postmortem [78]. This demonstrates the hardiness of the peripheral corneal epithelial cells as well as their regenerative potential. It was also observed that the number and viability of hLECs obtained was significantly lower for long-term stored corneal tissues [79]. This would suggest that the hLECs were eliminated during storage as mentioned previously during storage in organ culture [12] or in Optisol at 4 °C [80]. The key determining factor would again be the LSCs present in the tissues, which can also be affected by the storage conditions before their isolation [5,74]. Indeed, previous studies showed that stored corneal tissues generated cultures of hLECs [81,82] that proliferated more slowly [83] and were less likely to reach confluence [84] compared to those of unstored fresh tissues. As the mean ST was 24.77 days and the majority of adverse metabolic changes leading to apoptosis were reported during the first 2 weeks of storage [16,85,86], these observations seem normal. Other results showed that the LSC culture after 4 days of storage could not form a typical stratified epithelial structure, although the original corneal tissue showed a comparable level of expression of limbal stem/progenitor cell markers and an intact epithelial structure [29]. These different results are consistent with ours and indicate that the increase in storage time decreases the growth of hLEC cultures. We also observed that this increase in storage time was associated with a decrease in the percentage of stem cells associated with a decrease in the wound healing dynamic, as well as a decrease in the thickness of our reconstructed tissues for the majority of the hLEC populations concerned. Consistent with these other studies [29,85], and despite a reduction in expression compared to the SPM/ST condition, our _LPM/ST_hTECs still expressed the various corneal epithelial markers analyzed.

It is worth noticing that some populations belonging to the long PM/ST group presented features more related to hLECs from the short PM/ST group. This was especially the case for the 18-day population (_LPM/ST_hLEC-18), whose wound closure time was within the time frame of those of the short PM/ST group in our scratch wound assay. This result is consistent, since this same population also exhibited high values of doubling time and colony-forming efficiency. As to why the _LPM/ST_hLEC-18 behaved more like _SPM/ST_hLEC-1 and _SPM/ST_hLEC-2 is obviously not related to either the age of the patient, as the _LPM/ST_hLEC-18 hLECs were isolated from a patient in the same group of age as those with a long PM/ST (74-, 64-, 65- 67-, and 75-years old for _LPM/ST_hLEC-3, -4, -15, -17, and -18, respectively), nor from the cause of death (the patient for _LPM/ST_hLEC-18 died of infarction, as for patients with long _LPM/ST_hLEC-4 and -19 (Supplementary Table S1)). We rather believe that the features typically exhibited by _LPM/ST_hLEC-18 can be accounted for by interindividual variability. Indeed, several populations of hLECs presenting the same PM/ST could be expected to show different behaviors once cultured. This variation can have resulted from the number of proliferating cells (LSC and TAC) initially present in the donor corneas. This number varies between individuals, influenced in particular by the quality of the ocular surface in general at the time of the donor’s death [28]. Here, we can assume that the donor corneas from the 18-day population exhibited a larger than average proliferating cell pool. This could explain why this population behaved like _SPM/ST_hTECs even with an ST of 17 days. Nevertheless, our actual groups still showed significant differences for the proliferative properties, the percentage of stem cells, and the wound healing kinetics, and were in agreements with the results of the transcriptomic and PCA analyses, which clearly demonstrated that the _SPM/ST_hLEC-0, -1, and -2 populations were distinct from the remaining populations (_LPM/ST_hLEC-3, -4, -14, -15, -17, -18, and -19).

CEBPD is part of a highly conserved family of transcription factors expressed in different cell types and involved in proliferation control, cell differentiation, metabolism, and inflammation [87,88]. Our gene profiling analyses showed that its expression was increased in hLECs with a short PM/ST. Barbaro et al. showed that CEBPD and Bmi-1 could be good predictors to identify quiescent stem cells in the human limbus [50]. CEBPD is known to induce G0/G1 cell cycle failure in epithelial cells [50,89,90]. CEBPD is expressed in about 10% of basal limbal epithelial cells and its expression decreases according to cell proliferation [50]. The authors showed that CEBPD allowed the mitotic quiescence of LSC by the positive regulation of p27 and p57 [50,91,92,93], while preserving their proliferative potential by maintaining the expression of ΔNp63α [50,94]. Our Western blot analyses confirmed the variations observed at the transcriptomic level and showed a higher CEBPD protein expression in _SPM/ST_hLECs. Therefore, the _SPM/ST_hLECs condition better preserved stem cells within the hLECs cultures while they also simultaneously maintained their proliferative potential. This explains why _SPM/ST_hLECs had a higher percentage of stem cells and could proliferate faster in culture. Therefore, a higher level of CEBPD in hLECs cultures could allow a better maintenance of stem cells in the hTECs epithelium. Meanwhile, a greater proportion of stem cells in _SPM/ST_hLECs could also explain their ability to heal faster in our wound healing assays.

Another interesting observation is the increased expression of the *PFKL* gene in _SPM/ST_hLECs compared to _LPM/ST_hLECs in our microarray analysis, which was further confirmed at the protein level. Phosphofructokinase 1 (the product of the *PFK-1* gene) catalyzes the first irreversible reaction of glycolysis, making it an important part of this highly regulated process [95]. Indeed, p53, known as a tumor suppressor, can prevent tumor growth by suppressing *PFK-1* activity, therefore impeding cell proliferation [96,97]. The inhibition of *PFKL* transcription in a pulmonary epithelial cell line has been shown to result in decreased glycolysis and cell growth [98]. This indicates that *PFKL* can be important in the normal function of PFK-1 and therefore in the proper course of glycolysis. 

The phosphoinositide 3-kinases (PI3K) signaling pathway constitutes another major regulator of glucose metabolism. PI3K signaling via the AKT kinase can increase glucose absorption by increasing GLUT1 glucose transporter expression [99,100,101,102], a pathway that can be activated in response to growth signals [103,104]. In cancer, the activation of the PI3K pathway appears to be an important way to increase hypoxia-inducible transcription factors 1 (HIF-1) that, in turn, accounts for the increased expression of glycolytic enzymes [104,105,106,107]. These results therefore indicate that the PI3K pathway is important for ensuring proper glycolysis occurs. Our data showed that *GPR75* expression was also increased in _SPM/ST_hLECs at both the transcriptional and protein levels. GPR75 is a G-protein-coupled receptor (Gq) that has two known ligands, CCL5 and 20-HETE [108,109,110,111,112]. These two ligands are associated with different metabolic processes involved in cardiovascular diseases, glucose homeostasis, and obesity [109,111,113]. As the activation of GPR75 stimulates PI3K signaling, it is very likely it will also result in an increased glycolysis in hLECs.

In our study, we also noted that the expression of the *FZD9* gene was also increased in _SPM/ST_hLECs. Myc is a highly pleiotropic transcription factor that regulates cell expansion by coordinating various cellular processes, including proliferation, metabolism, and cell growth [114]. While Myc expression has traditionally been placed upstream of the Wnt signaling pathway, some studies have indicated the opposite [115,116]. Indeed, it has been shown that the Wnt FZD9 pathway receptor, encoded by the *FZD9* gene, was involved in a positive feedback loop involving the Myc and Wnt signaling pathways. Myc deregulation improves Wnt signaling by increasing the regulation of FZD9, which could, at the same time, promote the overexpression of Myc. Therefore, Myc could be a downstream target of Wnt [57]. FZD9 is also positively regulated in several types of cancer [117,118,119] and its inhibition leads to a decrease in cell proliferation and motility [120]. Moreover, it has been shown that Myc promotes the transcription of glucose transporters and glycolytic enzymes [121,122], which means that the FZD9 action on Myc would potentially increases glycolysis. 

Our data suggested that hLECs with a short PM/ST increased the expression of various factors whose encoded products promote glycolysis, which was consistent with the fact that the _SPM/ST_hLECs had a higher proliferative capacity than that observed for _LPM/ST_hLECs. A more active glycolysis also suggested that these cells were metabolically more active, also explaining why they better proliferated in culture. Our gene profiling and IPA analyses supported this hypothesis as they revealed an increase in the expression of several genes involved in the regulation of cell proliferation for the _SPM/ST_hLECs group.

A principal component analysis of transcriptomic profiles of the hLECs populations revealed that a change in gene expression arose from PM/ST 3, which prompted us to define our experimental groups (PM/ST 0, 1, 2 days vs. PM/ST 3 days and more). These observations were correlated with an increase in storage time. In fact, all corneas were collected within 24 h following the donor’s death. All of them were then stored in corneal viewing chambers at 4 °C, except for _SPM/ST_hLEC-1. From _LPM/ST_hLEC-3, which corresponded to 2 days of storage, the gene expression profiles became uniform and similar to that of _LPM/ST_hLEC-19 that was stored for 18 days. According to the Arrhenius relation [123], the hypothermic storage induces a decrease in cellular metabolism, that can therefore explain the general decrease in cell transcription activity. Furthermore, deleterious effects of cell cooling exist, limiting their maximum storage time, no matter the cell type [124]. Indeed, and consistent with both the decreased proliferation and the lower holoclone percentage of _LPM/ST_hLECs noted in the monolayer culture, the cold could cause a loss in the maintenance of limbal stem cells within the preserved tissues [125]. The rapid growth of these cells, once cultured in vitro, could have resulted from the maintenance of a more active cellular metabolism characterized by the upkeep expression of genes involved in the regulation of glycolysis. The increase in the expression of genes, whose protein products participate in the cell proliferation, could have occurred as a normal process for _SPM/ST_hLECs that had not been conditioned by hypothermic storage, which was supported by our functional analyses of the transcriptional profiles. In silico directional prediction of biological processes of interest, such as the proliferation of epithelial cells, colony formation, wound healing, and proliferation of stem cells revealed that the mean gene expression profile of _SPM/ST_hLECs would likely promote stem cell maintenance and cell proliferation when compared to _LPM/ST_hLECs. These analyses are quite consistent with the fact that _SPM/ST_hLECs and _SPM/ST_hTECs displayed an increased wound healing response in our wound closure experiments. 

Corneal tissue storage methods were designed primarily for the preservation of the corneal endothelium. It is therefore possible to make modifications to this protocol in order to promote the survival of corneal epithelial stem cells. For example, it would be possible to change the storage medium during the storage period that is up to one month. During this time, the various components of the medium are depleted, and waste products accumulate, causing cell-damaging changes, such as intracellular edema [80]. As detrimental metabolic changes [86] and cell death [16] occur early, it may be that much shorter storage periods, regular medium changes, or different media would improve hLECs survival. Reducing the PMT can also improve the general state of the epithelium at the beginning of storage. Further research into alternative storage methods for corneal epithelia would therefore be beneficial. Moreover, a study showed that storing corneal tissue in Optisol at 4 °C at the air–liquid interface resulted in a healthier epithelium with increased cell proliferation, higher expression of stem cell markers, and less cell death [126]. If the corneal tissue is to be used for keratoplasty and epithelial cell transplantation therapy, a compromise in storage techniques must be sought.

Despite the limitations of this study, we provided evidence that fresh tissues (small PMT and ST) are more likely to provide hLECs of higher quality. This would ultimately lead to an increase in the success of cell therapies in the clinic.

## 5. Conclusions

Our results suggested that the transcriptomic profile of hLECs was more uniform from a PM/ST of 3 days and beyond compared to a PM/ST of 0, 1, and 2 days, although care must be taken in their interpretation as they resulted from the analysis of three different populations of HLECs for the short PM/ST. This change caused a decrease in the expression of genes involved in cell proliferation, differentiation, and colony formation. In the hTEC, this change deeply impacted on the epithelium thickness and the wound healing dynamic. Consequently, for the conception of reconstructed tissues intended to be grafted for the treatment of patients with LSCD, but also for in vitro preclinical studies, it would clearly be more appropriate to use cells with a short PM/ST. More importantly, we believe these results are not exclusive to the cornea but could be valid for other epithelial tissues that also host stem cells. 

## Data Availability

All microarray data presented in this study comply with the *Minimum Information About a Microarray Experiment* (MIAME) requirements (GEO# GSE185459).

## Supplementary Materials

The following supporting information can be downloaded at: https://www.mdpi.com/article/10.3390/cells11172716/s1, Figure S1: Ingenuity pathway analysis (IPA) of gene interaction networks altered by PM/ST of hLECs, Figure S2: Morphology of hLECs with short or long PM/ST. Table S1: hLECs populations used in the study.
