# Supplementary material for: Influence of the Postmortem/Storage Time of Human Corneas on the Properties of Cultured Limbal Epithelial Cells
Source: Cells. 2022 Aug 31;11(17):2716. doi: 10.3390/cells11172716 (PMC9455001; doi:10.3390/cells11172716)
Supplement: Supplementary file 1 [file cells-11-02716-s001.zip › cells-1776961-supplementary.pdf]

# *Supplementary Materials*

## Influence of the post-mortem/storage time of human corneas on the properties of cultured limbal epithelial cells

Gaëtan Le-Bel,[1,2,3,4,a] Pascale Desjardins,[1,2,3,4,a] Christelle Gross,[2,4] Sergio Cortez Ghio,[1,3] Camille Couture,[1,2,3,4] Lucie Germain[1,2,3,4] and Sylvain L. Guérin[1,2,3,4*]

[1]Laboratoire d'organogénèse expérimentale (LOEX), Centre de Recherche du CHU de Québec - Université Laval, Axe Médecine Régénératrice, Québec, QC G1J 1Z4, Canada

[2]Centre Universitaire d'Ophtalmologie (CUO)-Recherche, Centre de recherche du CHU de Québec - Université Laval, Axe Médecine Régénératrice, Québec, QC G1S 4L8, Canada

[3]Département de Chirurgie, Faculté de médecine, Université Laval, Québec, QC G1V 0A6, Canada

[4]Département d'Ophtalmologie, Faculté de médecine, Université Laval, Québec, QC G1V 0A6, Canada

[a]Equally contributed as first authors

*Correspondence: Sylvain.Guerin@fmed.ulaval.ca; Tel.: +1-418-682-7565

**Supplementary Table S1.** hLECs populations used in the study

| Population name | Age of donor | Sex of donor | Cause of death | Date of death | Date of sampling | Post-mortem time | Date of cell extraction | Storage time | PM/ST |
|---|---|---|---|---|---|---|---|---|---|
| PPGG1884X | 84 | W | Infarction | 25/07/2018 | 25/07/2018 | 0 | 25/07/2018 | 0 | 0 |
| PPIJ1776X | 76 | W | Infarction | 23/10/2017 | 24/10/2017 | 1 | 24/10/2017 | 0 | 1 |
| PPJK1773X | 73 | W | Pulmonary embolism | 22/11/2017 | 23/11/2017 | 1 | 24/11/2017 | 1 | 2 |
| BYPL1774Y | 74 | M | Lung neoplasm | 16/12/2017 | 17/12/2017 | 1 | 19/12/2017 | 2 | 3 |
| BYFA1864X | 64 | W | Infarction | 26/01/2018 | 27/01/2018 | 1 | 30/01/2018 | 3 | 4 |
| BYTA1857X | 57 | W | Lung neoplasm | 12/01/2018 | 13/01/2018 | 1 | 26/01/2018 | 13 | 14 |
| BYQA1865Y | 65 | M | Bladder neoplasm | 15/01/2018 | 16/01/2018 | 1 | 30/01/2018 | 14 | 15 |
| BYBL1767X | 67 | W | Subarachnoid hemorrhage | 30/12/2017 | 31/12/2017 | 1 | 16/01/2018 | 16 | 17 |
| BYEEL1775Y | 75 | M | Infarction | 01/12/2017 | 02/12/2017 | 1 | 19/12/2017 | 17 | 18 |
| BYBL1757X | 57 | W | Infarction | 30/12/2017 | 31/12/2017 | 1 | 18/01/2018 | 18 | 19 |

The post-mortem/storage time (PM/ST) is the sum of both the post-mortem and storage time.
W: woman, M: man

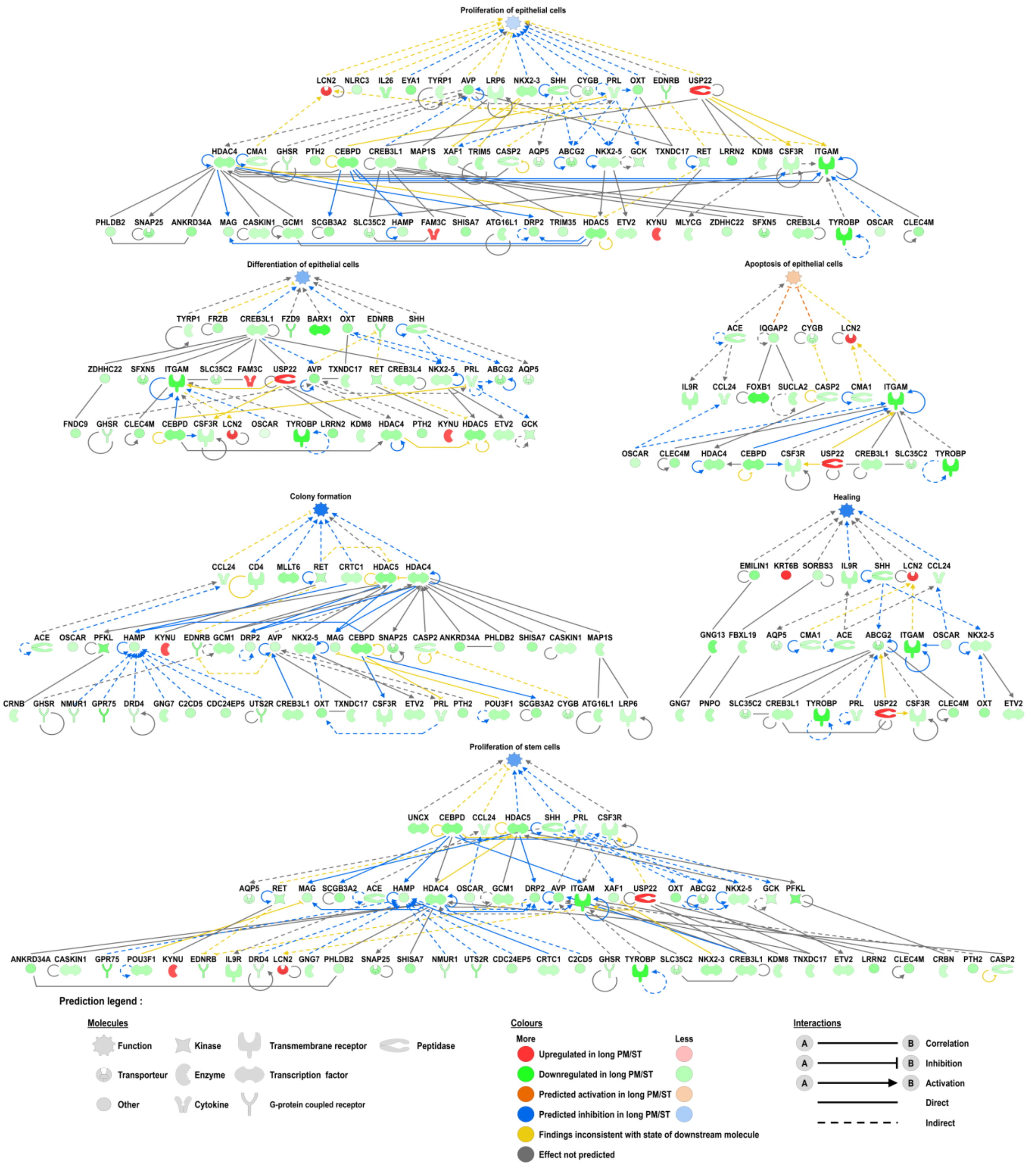


**Supplementary Figure S1. Ingenuity pathway analysis (IPA) of gene interaction networks altered by PM/ST of hLECs.**

Gene interaction networks altered by $_{SPM/ST}$hLECs or $_{LPM/ST}$hLECs built around biological functions of interest: proliferation, differentiation and apoptosis of hLECs, and colony formation, healing and stem cells proliferation. Differentially expressed genes present in our datasets are indicated and colored either green or red depending on whether they were respectively up- or down-regulated, relatively to hLECs from the short PM/ST group. Lines indicate gene-gene and gene-function relationships (full and dotted lines for direct and indirect relationships, respectively) based on IPA's database.

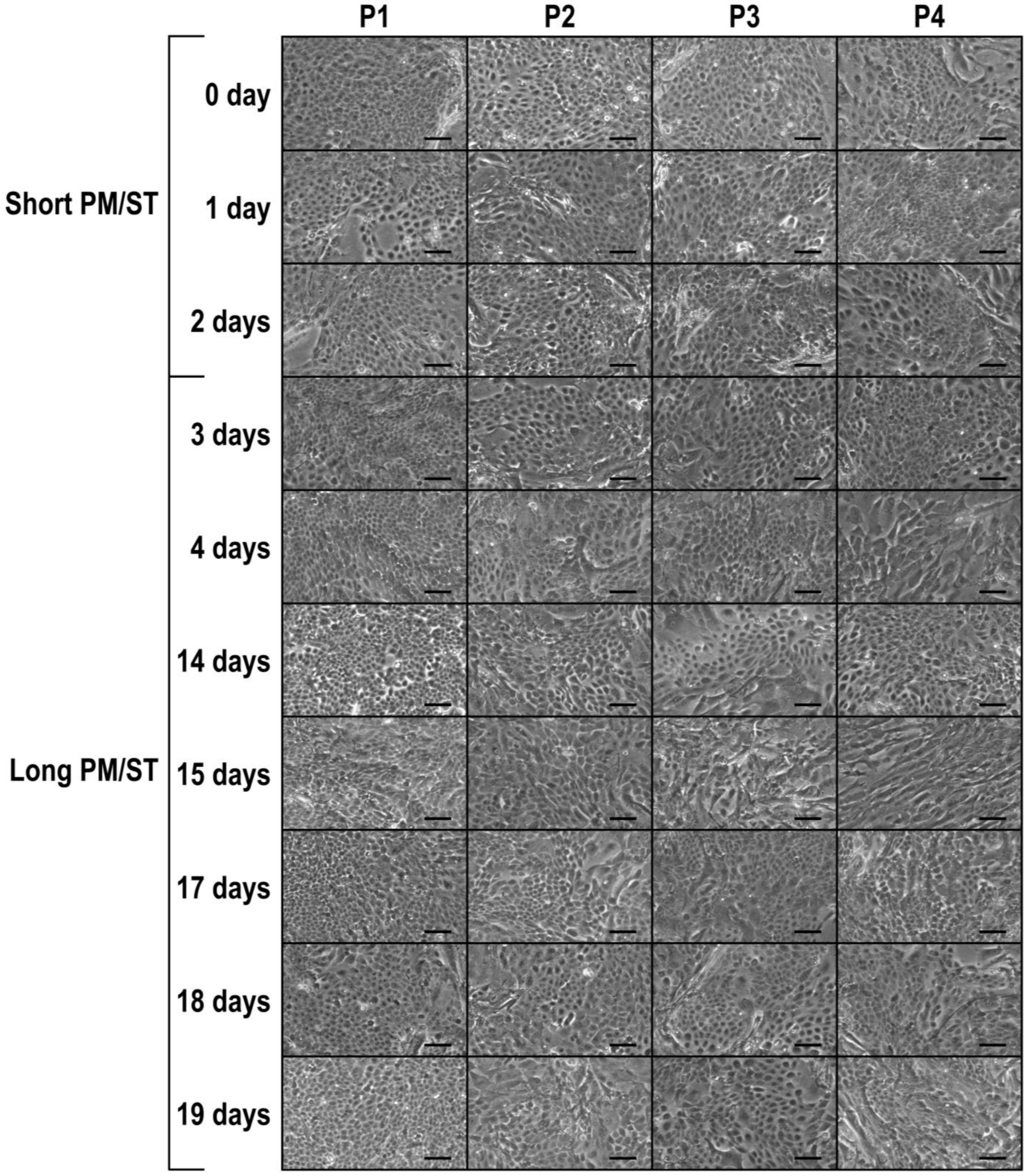


**Supplementary Figure S2. Morphology of hLECs with short or long PM/ST.**
Morphology by phase contrast microscopy of hLECs with either short or long PM/ST cultured at passage P1 to P4. Scale bars: 200 µm.
